# Enhancement of RecA-mediated self-assembly in DNA nanostructures through basepair mismatches and single-strand nicks

**DOI:** 10.1038/srep41081

**Published:** 2017-01-23

**Authors:** Sybilla Louise Corbett, Rajan Sharma, Alexander Giles Davies, Christoph Wälti

**Affiliations:** 1 School of Electronic and Electrical Engineering, University of Leeds, Leeds, LS2 9JT, U.K

## Abstract

The use of DNA as a structural material for nanometre-scale construction has grown extensively over the last decades. The development of more advanced DNA-based materials would benefit from a modular approach enabling the direct assembly of additional elements onto nanostructures after fabrication. RecA-based nucleoprotein filaments encapsulating short ssDNA have been demonstrated as a tool for highly efficient and fully programmable *post-hoc* patterning of duplex DNA scaffold. However, the underlying assembly process is not fully understood, in particular when patterning complex DNA topologies. Here, we report the effect of basepair-mismatched regions and single-strand nicks in the double-stranded DNA scaffold on the yield of RecA-based assembly. Significant increases in assembly yield are observed upon the introduction of unpaired basepairs directly adjacent to the assembly region. However, when the unpaired regions were introduced further from the assembly site the assembly yield initially decreased as the length of the unpaired region was increased. These results suggest that an unpaired region acts as a kinetic trap for RecA-based nucleoprotein filaments, impeding the assembly mechanism. Conversely, when the unpaired region is located directly adjacent to the assembly site, it leads to an increase in efficiency of RecA patterning owing to increased breathing of the assembly site.

DNA nanotechnology has become established as a successful means to create a variety of structures and devices on the nanometre scale, and many sophisticated nanostructures made entirely from DNA have now been demonstrated. The growth of the field has been enabled by a number of breakthroughs including the development of: branched DNA junctions^1^, one-dimensional tracks^2^, higher-order periodic and aperiodic lattices^3^, programmable nanoarrays^4^, three-dimensional polyhedra^2^,^5^, DNA origami^1^,^6^, two and three-dimensional constructs using origami^2^,^7^,^8^,^9^, and the fabrication of micrometre-size crystals^3^,^10^ with precisely controlled depths^4^,^11^, *inter alia*. However, the potential of DNA-based nanotechnology for dynamic applications is hampered by current approaches to structure augmentation – it is currently difficult to control or adjust the attachment of additional components onto structures in a flexible and programmable way once a DNA scaffold has been made. Current strategies to assemble further components onto DNA nanostructures require either short single-stranded (ss)DNA anchors or nucleic acid aptamers to be designed into the DNA scaffolds to allow subsequent assembly of further oligonucleotides^2^,^5^,^12^,^13^,^14^, or other inorganic or organic molecules^15^,^16^, respectively, or the inclusion of specific nucleotide base sequences in the scaffolds as docking sites for chemical intercalation^17^,^18^, for example. Although these strategies are functional, they are not versatile as they all require the scaffolds to be redesigned every time the type and local arrangement of the elements on the DNA structure need to be changed. Hence, flexible and programmable assembly mechanisms are desirable and would represent a considerable extension of DNA nanotechnology.

The scope of DNA nanotechnology would also be extended if it were combined with other biological molecules that also possessed intrinsic self-assembly properties. DNA-binding proteins are ideally placed to work in conjunction with DNA-based nanostructure technologies; an example includes the *E. coli* protein Recombinase A (RecA), which has been exploited as a tool for highly efficient and fully programmable patterning of DNA scaffolds^19^,^20^,^21^,^22^. RecA proteins can polymerise onto ssDNA to form helical nucleoprotein filaments that can be assembled site-specifically and with single base-pair resolution anywhere onto double-stranded (ds)DNA scaffolds (Fig. 1). Nucleoprotein filaments as short as 3 nm, i.e. made from ssDNA just six nucleotides long, have been demonstrated to pattern dsDNA effectively^23^.

Central to this site-specific assembly technique is the process of homologous recombination that is catalysed by the RecA family of ATPases, and involves exchange of strands between two DNA molecules featuring regions with matching nucleotide sequence^24^,^25^. In the presence of magnesium ions^26^ and a nucleotide co-factor^27^, RecA binds to a ssDNA molecule with a stoichiometry of three nucleotides per protein monomer to form a right-handed helical nucleoprotein filament, which is reminiscent of one half of the relaxed B-form of DNA. In this form there are approximately 6.2 RecA monomers (or 18 nucleotides) per helical turn and the complex has a diameter of approximately 10 nm^28^,^29^,^30^. Nucleoprotein filament formation is initiated through the nucleation of 4–5 monomers of RecA protein onto a ssDNA molecule. This is followed by a highly cooperative rapid extension phase in which monomers of RecA protein are added primarily in the 5′ to 3′ direction with respect to the ssDNA^31^. The activity of the nucleoprotein filament depends on the nucleotide cofactor^32^,^33^: in the presence of ATP or ATPγS (a non-hydrolysable analogue), the nucleoprotein filaments assume an enzymatically active extended state with a helical pitch of 95 Å capable of interacting with dsDNA and catalysing homologous strand exchange reactions^27^, whereas functionally inactive compressed filaments with approximately five nucleotides per RecA monomer (helical pitch 75 Å) are formed in the absence of ATP or ATPγS^34^,^35^.

During homologous recombination, the active nucleoprotein filament binds non-specifically to one of the strands of an incoming dsDNA molecule through a weaker secondary site to form an unstable three-stranded intermediate complex^36^. The formation of this homology probing complex requires local melting of the incoming dsDNA to make the individual strands available. However, the distance between the primary and secondary binding site in RecA is of the order 25 Å^28^ and thus fewer basepairs will be formed between the constituent ssDNA of the nucleoprotein filament and the ssDNA of the incoming dsDNA than were broken to melt locally the incoming dsDNA^36^. Furthermore, the affinity of the secondary binding site to ssDNA has been shown to be relativly weak. Therefore, it has been suggested that the initiation of the homology probing relies on the spontaneous formation of DNA bubbles, i.e. a spontaneous and transient breakage of one or few basepairs, a phenomena generally referred to as ‘breathing’, and a sizeable body of work has been reported in support of this model^36^,^37^,^38^,^39^.

Once this intermediate homology probing complex is established, it then searches the dsDNA for a region with a sequence homologous to that of the encapsulated ssDNA. This occurs through a combination^37^ of reversible thermally-driven random sampling via heterologous association^40^, intersegmental contact sampling^41^, and short range (60–300 bp) one-dimensional facilitated diffusion^42^. Both DNA molecules, the double-stranded scaffold as well as the ssDNA encapsulated in the nucleoprotein filament, remain part of the triple-standed complex. FRET studies have suggested that the propagation of the homology search occurs in three-basepair-steps^43^,^44^.

When homologous alignment is established, all Watson-Crick basepairing hydrogen bonds of the dsDNA within the homologous triple-standed nucleoprotein complex are broken, and new hydrogen bonds between the constituent ssDNA of the RecA-based filament and its complementary strand on the dsDNA molecule are formed, resulting in the exchange of the homologous strands^37^,^45^. Subsequently, ATP bound to the RecA proteins hydrolyses, which causes a conformational change in the protein and, consequently, leads to the disassembly of the nucleoprotein complex. However, the triple-stranded nucleoprotein complex remains intact if a non-hydrolysable variant of ATP, ATPγS, is used instead^46^,^47^. It is the ability of RecA-based nucleoprotein filaments to form stable triple-stranded complexes that has potential application in DNA and molecular nanotechnology as a programmable assembly tool.

Previous investigations of RecA-driven triple-stand formation have focussed on linear and circular dsDNA templates^23^,^48^,^49^. However, in the context of DNA nanotechnology, the double-stranded DNA scaffolds generally comprise more complex topologies such as non-basepaired regions, and the effect of such structures on the RecA-based assembly yield is not yet understood.

Here, we report the effect of the introduction of basepair mismatches and single-strand nicks on the yield of RecA-based nucleoprotein filament assembly on a dsDNA scaffold. Significant increases in yield were observed upon the introduction of unpaired regions – reminiscent of permanent DNA bubbles – directly adjacent to the assembly region. However, a more complex behaviour was found when short unpaired regions were introduced away from the assembly site, where, as the length of the unpaired region was increased, the yield initially decreased, and then increased. These results suggest that an unpaired region in general interferes with the search mechanism of the RecA nucleoprotein filaments, although when located directly adjacent to the assembly site, it conversely enhances access to the homologous region and thus binding of the RecA filament. We propose that this enhanced access is enabled by increased breathing of the homologous site resulting from being positioned directly adjacent to the unpaired region. This hypothesis is corroborated by the introduction of single-strand nicks in the dsDNA adjacent to the assembly site, which also enhances breathing in the vicinity of the nick and thus also provides greater access to the DNA scaffold for the RecA nucleoprotein filament.

## Results and Discussion

**Figure 2:**
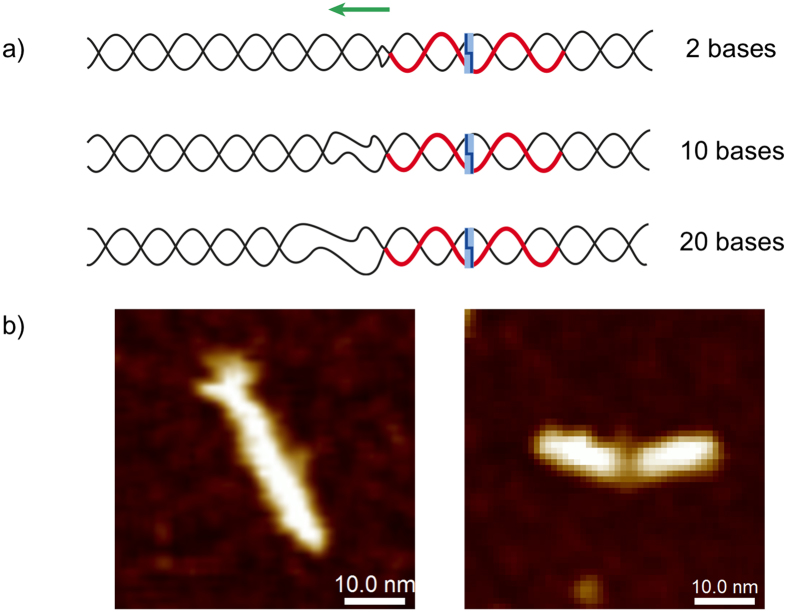
(**a**) Schematic illustration of a series of 100 basepair dsDNA structures featuring basepair-mismatched regions. Each dsDNA scaffold contains a nucleoprotein filament assembly site located adjacent to the mismatched region. The extension of the unpaired region between scaffolds is in the 3′ to 5′ direction (indicated by the green arrow) i.e. away from the patterning region. The nucleoprotein filament assembly site is indicated in red and the recognition site of the XapI enzyme is shown as a blue box. (**b**) Peak force tapping AFM image of a 100 bp DNA molecule with fully basepaired matching sequence (left) and a 100 bp DNA molecule containing a 20-basepair unpaired region located directly adjacent to the nucleoprotein filament assembly site (right). The height profiles for both DNA molecules, taken along the long axis of the molecule, are shown in Figure S2.

Double-stranded DNA scaffolds were generated from synthetic 100-basepair DNA oligomers containing a 30-basepair region homologous to the sequence of the single-stranded DNA of the RecA nucleoprotein filaments, and featuring basepair mismatched regions of various lengths directly adjacent to the nucleoprotein assembly site. Figure 2a shows a diagrammatic representation of the design of the various DNA scaffolds employed. The assembly site (indicated in red) is located between basepairs 61–90 and also features a recognition site for the restriction enzyme XapI (basepairs 68–73; shown as a blue box in Fig. 2a). The basepairing is interrupted adjacent to the nucleoprotein filament assembly site as indicated. The single-stranded DNA oligomers were assembled into double-stranded scaffolds by annealing and the products, containing mismatches of 0, 2, 6, 8, 10, 12, 15 and 20 bases, were analysed by PAGE (Supplementary Figure S1). The double-stranded scaffold with no or only small mismatched regions run at approximately 100 basepairs as expected, while increasing the length of the mismatched region reduces the mobility of the DNA through the gel, indicating that significant structural differences exist between the different constructs. Figure 2b shows representative atomic force microscopy (AFM) images of the two extreme cases, i.e. the fully basepaired scaffold (left) and the scaffold featuring a 20-basepair mismatched region directly adjacent to the assembly site. The images reveal a distinct structural difference between the two constructs. The height profiles taken along the long axis of the DNA scaffolds are shown in Supplementary Figure S2.

**Figure 3:**
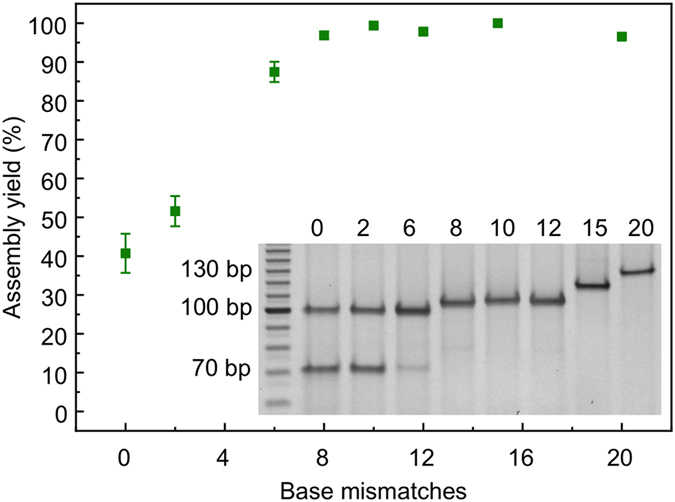
Densitometry analysis showing the assembly yield of a 30-nucleotide-long nucleoprotein filament onto 100 bp dsDNA scaffolds containing mismatches of 0, 2, 6, 8, 10, 12, 15 and 20 bases. The patterning site is located directly adjacent to the mismatched region of the scaffold. The assembly yield was obtained via XapI enzyme digestion assays. The data represent the average of three independent experiments and the error bars show standard error of the mean. The inset shows a representative 15% PAGE gel (19:1, 80 V, 360 min) showing the results of a restriction enzyme assay following nucleoprotein filament assembly. A single band indicates no digestion. In lanes with two bands, the lower band corresponds to a DNA fragment resulting from the XapI enzyme digestion (with a further DNA fragment running at approximately 30 bp and not visible on the gel image). The number of bases that are not basepaired is indicated above the lanes on the gel. The bands in the first lane represent a 10 bp DNA ladder.

The yield of RecA-based nucleoprotein filament assembly can be assessed indirectly through a restriction assay, where the recognition site of the enzyme lies within the triple-stranded region^23^,^50^. When the triple-stranded nucleoprotein complex is formed successfully, the access of the restriction enzyme to the restriction site on the double-stranded scaffold is obstructed thus preventing digestion; hence the restriction efficiency can be used to estimate the assembly yield^21^. Here, the effect of the basepair mismatched regions on the assembly yield of RecA-based nucleoprotein filaments formed from a 30-nucleotide-long ssDNA onto dsDNA scaffolds was investigated using a XapI restriction assay. A PAGE gel showing the results of the XapI restriction assay is shown in the inset to Fig. 3. For the fraction of the scaffold DNA where the assembly of the nucleoprotein filament was successful, the scaffold DNA is not expected to be digested. As such, a band on the gel, running at approximately 100 bp for no or short mismatched regions, and slightly longer for longer mismatched regions (see also Supplementary Figure S1), is expected to be observed. For the fraction of DNA scaffolds where assembly is not successful, two bands representing the two digestion products are expected, running at approximately 30 bp (not visible on the gel image) and 70 bp for no or short mismatches or slightly longer for increasing size of mismatched regions. From the inset to Fig. 3 it can be seen that the intensity of the band representing the undigested scaffold increases with increasing number of mismatches, while the intensity of the band representing the digested products decreases with mismatches, suggesting that the assembly yield increases with increasing mismatches.

The assembly yields, calculated from the relative intensities of the two bands, are shown in the main panel of Fig. 3. The data represent the average of three experiments and the error bars represent the standard error of the mean. For no mismatches, an assembly yield of approximately 40% is observed, which is in broad agreement with previous observations of RecA-mediated patterning under similar conditions^23^. The yield saturates at around a mismatch region length of 8 basepairs, above which yields of close to 100% are observed. A range of control experiments were carried out to demonstrate that the inhibition of scaffold digestion was indeed due to RecA-mediated assembly of the nucleoprotein filament onto the scaffold using heat-inactivated RecA and by omitting the restriction enzyme (Supplementary Figure S3a,b). We note that when using heat-inactivated RecA full digestion was observed, confirming that the reduction in digestion efficiency is caused by successful RecA-mediated assembly of the nucleoprotein filaments and thus protection of the restriction site (Supplementary Figure S3a). Furthermore, no digestion was observed when the same assay was carried out but omitting the restriction enzyme (Supplementary Figure S3b).

Double-stranded DNA molecules experience local thermal fluctuations where the hydrogen bonds between one or more adjacent complementary bases are transiently broken and thus lead to an unstacking of the bases at temperatures well below the melting temperature of the DNA. Such DNA breathing, which leads to local transient DNA bubbles and thus exposure of ssDNA sections which are otherwise buried in the duplex, underpins the interaction mechanism of many DNA binding proteins^51^. For example the binding of the human transcription factor YY1 to dsDNA in cells is correlated with the propensity of breathing of the dsDNA^38^. Importantly for this work, the homology probing of the recombinase proteins of the recombinase A superfamily, including RecA, has been reported to be initiated by DNA breathing^37^. However, only a very small fraction of the dsDNA is affected at any one time by the formation of DNA bubbles owing to internal breathing. In contrast, breathing is significantly enhanced at the ends of dsDNA, and indeed is even more pronounced at forked DNA junctions, e.g. where dsDNA is terminated with frayed ends, where even larger and longer-lived DNA bubbles exist^52^.

The pronounced increase in assembly yield upon introduction of unpaired regions adjacent to the assembly site suggests that these unpaired regions lead to enhanced breathing of the dsDNA at the termini of the unpaired region and thus boost access of the nucleoprotein filament to the assembly site^53^,^54^,^55^,^56^. The transient unpairing of the bases within the homologous region is reducing the energetic cost of formation of the initial homologous interactions between the nucleoprotein filament and dsDNA. Once an initial portion of the nucleoprotein filament is bound it becomes energetically favourable for further triplets to bind where there is sequence homology^57^.

**Figure 4:**
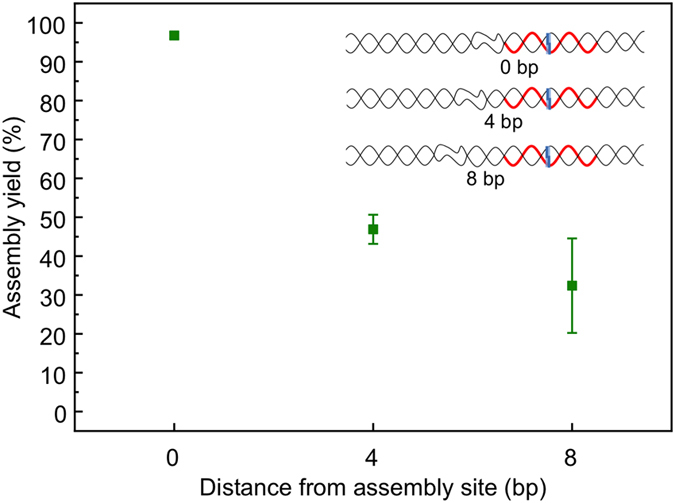
Densitometry analysis showing the effect of proximity of basepair mismatches from the assembly site on the efficiency of RecA nucleoprotein filament-mediated assembly. The inset shows a schematic illustration of the three different 100 bp DNA scaffolds used, each with 12-base-mismatched regions, located at 0, 4 and 8 bases from the nucleoprotein filament assembly site (red strand). Moving the 12-basepair-mismatched region away from the assembly site significantly decreases the yield of nucleoprotein-filament assembly. The data correspond to the average of three measurements, and the error bars show the standard error of the mean.

The position of the unpaired region on the dsDNA scaffold with respect to the assembly site is of critical importance. 12-basepair-long mismatches were found to be sufficient to achieve maximum assembly yield when the mismatched region was directly adjacent to the assembly site (Fig. 3). However, the assembly yield decreases significantly as the distance between the assembly site and the mismatched region is increased. Figure 4 shows the assembly yield estimated via the same restriction assay as above, for three different 12-basepair-long mismatched regions. The two additional 12-basepair-long mismatched regions were arranged such that one was positioned in the centre of the longest, 20-basepair mismatched region investigated above, i.e. 4 bp away from the assembly site, and the other at the far end of the 20-basepair mismatched region, i.e. 8 bp away from the assembly site. Surprisingly, the assembly yield for the scaffold with 8 bp distant region is even lower than the yield for the fully intact scaffold (approximately 30% *vs* 40%). This suggests that while a 12 bp mismatched region is sufficient to enable maximum assembly yield when directly adjacent to the assembly site, it impedes assembly when located away from the assembly site.

As discussed above, enhanced breathing of the dsDNA at the homologous assembly site is expected when unpaired regions are present immediately adjacent^52^. In contrast, as the end-breathing generally extends only a few basepairs into the dsDNA, an unpaired region further than this from the homology site is unlikely to enhance breathing at the homology site. The results suggest that although a small increase in assembly yield is still obtained for a four basepair separation between the unpaired region and the homology site, little benefit is observed when this distance is increased to eight basepairs where, in fact, a suppression of the assembly yield is observed.

It has been suggested that the process of homologous recombination involves one-dimensional facilitated diffusion over a range of several tens of basepairs of the intermediate homology probing complex^42^. We propose, based on our findings, that an extended unpaired region in the dsDNA interferes with this searching mechanism and thus leads to a reduction in assembly yield.

Where the region of increased breathing due to the DNA topology extends into the homology site, RecA binding is enhanced due to the lowered energetic cost of the initial binding event. When the unpaired region is not proximal to a region of homology, the initial binding of the nucleoprotein filament to the unpaired region is enhanced in a similar manner. However, where the DNA bubble, which led to the enhanced binding, was caused by thermal fluctuations, there is an energetic drive to close the bubble. Together with the fact that the affinity of the RecA-based nucleoprotein filament to the scaffold DNA is relativly weak when no homology is exists, this leads to a short lifetime of the non-homologous triple-stranded complex. In contrast, in the situation created here, the energetic drive to close the DNA bubble does not exist and the RecA-based nucleoprotein filament is likely to become kinetically ‘trapped’. We note that the topologies we have created are not likely to form *in vivo* except in the case of DNA damage, which RecA is recruited to repair; as a result it seems likely that the increased interaction of RecA with these structures is an aspect of RecA function on which possible selection pressure would be positive.

**Figure 5:**
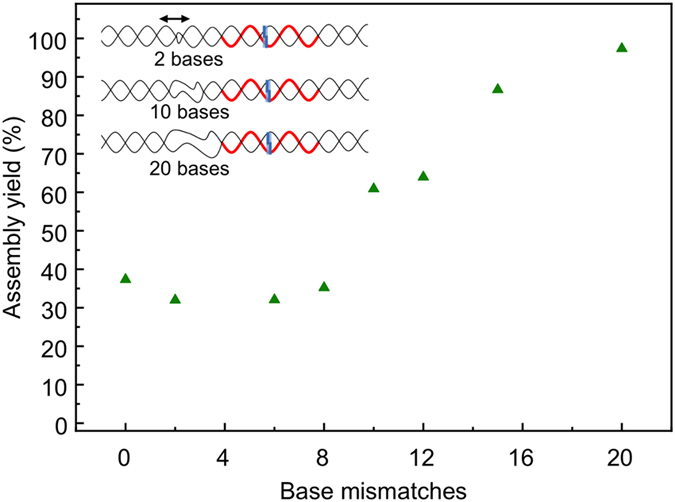
Yield of RecA-mediated sequence-specific assembly onto dsDNA scaffolds containing 0, 2, 6, 8, 10, 12, 15 and 20 basepair mismatches, each centered 10 bp away from the patterning site. The yield was obtained via an XapI enzyme digestion assay. The schematic diagrams of three of the different 100 bp dsDNA scaffolds used (containing 2, 10 and 20 bp mismatches, centered 10 bases from the nucleoprotein filament patterning region (indicated in red)) are shown in the inset.

A similar effect can be observed when considering scaffolds with different length mismatched regions (0–20 basepairs), but where, in contrast to Fig. 2, the mismatched regions are all centred 10 bp away from the assembly site. A schematic illustration of the scaffold design is shown in the inset to Fig. 5, and the assembly yields for the different scaffolds are shown in the main panel. The no-mismatch scaffold is identical to the one used in Fig. 2 and hence the assembly yield is the same. However, upon the introduction of short mismatches (2 basepairs), the yield decreases from 37% to 32%, which is in contrast to the results observed in Fig. 3, where even a 2-bp-mismatch led to a significant increase in assembly yield (52%). This again suggests that even the 2 bp-mismatched region, located 9 bp away from the assembly site, interferes with the searching mechanism of the RecA nucleoprotein filament on the double-stranded DNA scaffold owing to the kinetic trapping. A similar assembly yield was observed for scaffolds with 6-bp-mismatches, which suggests that although the assembly yield is improved by increasing the mismatched region as observed above, a longer mismatched region also increases interference with the searching mechanism. The positive impact on the assembly yield due to the increased mismatch, and decreasing distance between the mismatched region and the assembly site, increasingly outweighs the interference with the searching mechanism, and the sequence with a 10 bp mismatch shows a 60% assembly yield, which increases to more than 95% for the sequence with a 20 bp mismatch.

It has previously been shown that the ratio of nucleoprotein filaments to dsDNA scaffold has a significant influence on the assembly yield^21^. Here, while the assembly yields are lower for smaller ratios, the overall qualitative behaviour, with an initial decrease in yield followed by an increase as a function of mismatch length, is conserved for all assembling ratios investigated (Supplementary Figure S4).

**Figure 6:**
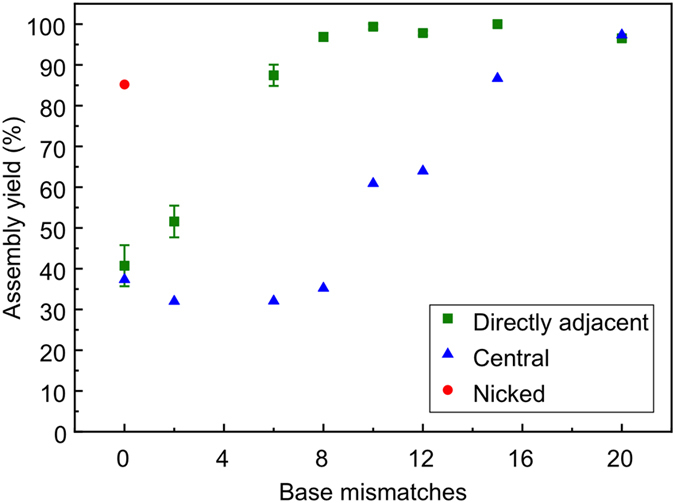
Comparison of assembly yield for basepair mismatched regions adjacent to (green squares) as well as away from (blue triangle) the assembly site. The red circle indicates the yield of assembly when using a fully basepaired dsDNA scaffold which was nicked immediately adjacent to the assembly site.

A nick in the phosphate backbone of one of the strands of the dsDNA adjacent to the assembly site leads to increased breathing in the vicinity of the nick and therefore is expected to lead to increased assembly yields of the RecA-based nucleoprotein filaments. Figure 6 shows the assembly yields for both types of scaffolds discussed above, together with the yield for a scaffold featuring a nick directly adjacent to the assembly site. Indeed, the assembly yield increases to over 85% upon introduction of the nick, providing further confirmation that these topologies are able to provide significant increases in patterning efficiency. Although significant, the increase in yield associated with a nick in the backbone is not as high as with regions of base pair mismatches proximal to the patterning site. It is likely that the hydrophobic effect and π-π interactions between the bases of the strand prevent the same level of breathing as would be observed at the terminus of a strand, and thus nicking only leads to smaller increases in patterning efficiency. The results of RecA-based nucleoprotein assembly onto scaffolds in which the position of the nick is varied is reported in Supplementary Figure S5a.

RecA-mediated assembly of nucleoprotein filaments is sequence-specific with the assembly sequence determined via the single-stranded DNA oligomer encapsulated in the nucleoprotein filament. For all results reported above, the short single-stranded DNA oligomer used to program the sequence-specificity of the nucleoprotein filament was homologous to the upper strand of the double-stranded DNA scaffold. However, very similar yields were obtained when using a reverse complementary oligomer, i.e. a single-stranded DNA that is homologous to the lower strand of the scaffold DNA. Supplementary Figure S5a,b shows such results when using scaffolds containing 12-base-mismatched regions located 0, 4 and 8 bases away from the nucleoprotein filament assembly site, as well as for all nicked scaffolds.

## Conclusion

We have demonstrated that RecA-based nucleoprotein filaments can successfully assemble site-specifically onto dsDNA scaffolds even when featuring complex topologies. We have shown that the yield of nucleoprotein filament assembly is strongly affected by non-linear features in the DNA scaffold. Non-basepaired regions adjacent to the assembly site greatly improved the yield of sequence-specific assembly, which increased from approximately 40% for fully basepaired dsDNA scaffolds to >95% upon the introduction of ≥10 unpaired nucleotides directly adjacent to the assembly site. However, a more complex behaviour was observed when a short non-basepaired region was introduced away from the assembly site, where an initial decrease followed by an increase in the filament assembly yield was observed with increasing length of the unpaired region. The results corroborate previously reported findings that the initiation of the homology probing relies on the spontaneous formation of DNA bubbles, which is significantly enhanced at the termini of dsDNA and in particular at forked DNA junctions. More importantly, our results demonstrate that the introduction of basepair mismatches distant from the homology site interfere with the assembly mechanism, most likely as the non-basepaired regions act as local kinetic traps for the RecA-based nucleoprotein filaments, and thus lead to a decrease in assembly yield.

These results provide a better understanding of the underlying mechanism of the RecA-based patterning of DNA nanostructures, and will find application in the construction of novel DNA-based heterogeneous nanomaterials, specifically to direct the assembly of additional components to DNA nanostructures once they have been made.

## Experimental Section

### Reagents

RecA protein from *E. coli* at a concentration of 2 mg/mL was obtained from New England Biolabs Inc. (Ipswich, USA). SYBR Gold Nucleic Acid Gel Stain, XapI enzyme (recognition sequence 5′…R^AATTY…3′, 10 units/μL) and 1X tango buffer containing tris acetate (TAc) (33 mM, pH 7.9), magnesium acetate (MgAc) (10 mM) potassium acetate (66 mM), and BSA (0.1 mg/mL) were purchased from Thermo Fisher Scientific Inc. (Waltham, USA). Proteinase K (>800 units/mL), ammonium persulphate, TAc, MgAc (1 M), tris-EDTA (TE) buffer (100X), N,N,N′,N′-tetramethylethylenediamine (TEMED) and acrylamide/bis-acrylamide (30%) were obtained from Sigma-Aldrich (St. Louis, USA). Synthetic oligomers were purchased from Integrated DNA Technologies (Leuven, Belgium) and were stored at a concentration of 100 μM in 1x TE buffer at −20 °C. Blue/Orange loading dye (6X) was obtained from Promega Corporation (Madison, USA).

### Generation of DNA scaffolds

The 100 base DNA sequence used in this work was obtained from the λ bacteriophage genome, where the 20 base non-basepaired sequence was designed and introduced to one of the strands such that it correctly anneals to its reverse complement at temperatures below 70 °C. Shorter mismatched regions were a section of the 20 basepair sequence, except where optimisation was necessary to facilitate correct basepairing.

To generate DNA scaffolds, the two complementary synthetic oligomers were mixed in equal quantities to a final concentration of 1 mM in a buffer containing Potassium Acetate (50 mM), Tris-acetate (20 mM), Magnesium Acetate (10 mM) and DTT (1 mM, pH 7.9). The solution was heated to 95 °C for 5 minutes, cooled by 2 °C per minute to 70 °C and then held at 4 °C. The efficiency of annealing was assessed by polyacrylamide gel electrophoresis (PAGE).

### Site-specific assembly of the nucleoprotein filament

The 30-nucleotide-long nucleoprotein filament used in this study was generated using the protocol reported previously^21^,^23^. Briefly, the 30-nucleotide-long synthetic oligonucleotide and RecA proteins were mixed at a concentration of one RecA protein per three nucleotides in a buffer containing TAc (30 mM) and MgAc (2 mM, pH 7.4) with ATPγS (20 mM), and the sample was incubated for 15 minutes at 37 °C. 30 base poly(T) (100 pmol) was then added to the sample to bind any excess functionally active unbound RecA, which could bind non-specifically to dsDNA. The mixture was incubated for an additional 15 minutes at 37 °C. Assembly of the nucleoprotein filaments were performed by adding DNA scaffolds to this reaction mixture at a concentration of between 1:10 and 1:40 of DNA to the nucleoprotein filaments in TAc (30 mM) and MgAc (10 mM), and incubating the sample at 37 °C for 45 minutes. Following the nucleoprotein filament assembly, the efficiency of the patterning was assessed using a restriction enzyme, where XapI (10 units) was added to the reaction mixture in Tango buffer (1X) and incubated at 37 °C for 5 minutes. Correctly assembled filaments would protect the dsDNA from enzymatic cleavage if the recognition site of the enzyme lies within the patterned region. The reaction was stopped by adding Proteinase K (≥4 units) and the mixture was incubated at 37 °C for 30 minutes. Following this step the DNA was visualised by PAGE to assess the efficiency of the nucleoprotein filament assembly by observing the protection from digestion afforded by the protein-DNA complex.

Control experiments were carried out using RecA denatured at 65 °C for 20 minutes. A non-hydrolysable ATP analogue, ATPγS, allows the formation of stable structures.

### Imaging and analysis

DNA was visualised by PAGE on polyacrylamide gels (15%, 19:1 acyrlamide/bis-acrylamide). Samples were loaded with Blue/Orange loading dye, run at 80 V for 360 minutes and stained with SYBR Gold Nucleic Acid Gel Stain. Imaging was carried out using Kodak 1D image analysis software.

Densitometry was undertaken using ImageJ. The total intensity in each lane was obtained and the relative intensity of each band as a percentage of the total lane intensity was calculated.

For analysing DNA scaffolds containing 0 and 20 base mismatches using AFM, 50 μl of DNA (8–10 ng in total) in 10 mM Tris-EDTA pH 8 buffer was allowed to adsorb on a freshly cleaved mica surface, pre-treated with 10 mM NiCl_2_ for 20 minutes in a high humidity chamber. A further 100 μl of 10 mM Tris-EDTA pH 8 buffer was afterwards added. The resulting sample was imaged with a Dimension FastScan Bio AFM (Bruker, USA) in peak force tapping mode, in liquid, with a Fastscan-D cantilever (Bruker, USA) at an amplitude set point of 250 mV and 57.68 mV drive amplitude. All the captured AFM images were analysed with the Nanoscope Analysis software from Bruker.

### Data Availability

Data associated with this work are available from the Research Data Leeds repository under a CC-BY license at http://dx.doi.org/10.5518/136.

## Additional Information

**How to cite this article**: Corbett, S. L. *et al*. Enhancement of RecA-mediated self-assembly in DNA nanostructures through basepair mismatches and single-strand nicks. *Sci. Rep.* **7**, 41081; doi: 10.1038/srep41081 (2017).

**Publisher's note:** Springer Nature remains neutral with regard to jurisdictional claims in published maps and institutional affiliations.

## Supplementary Material

Supplementary Information

## Figures and Tables

**Figure 1:**
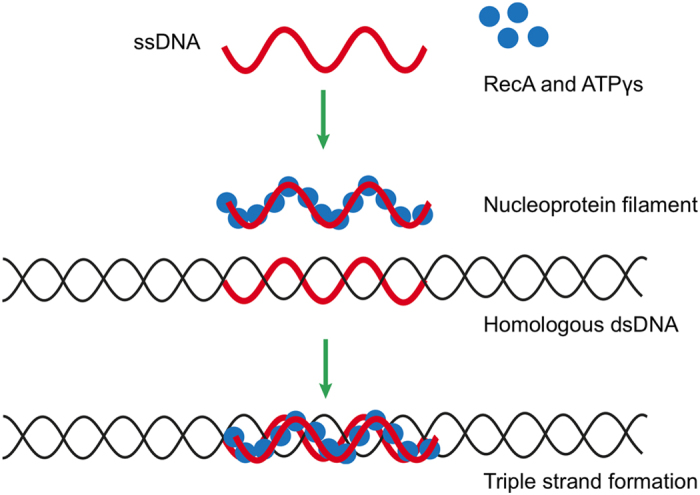
Schematic illustration showing the assembly of an active nucleoprotein filament onto a dsDNA scaffold. A triple-stranded complex is formed at a region of the dsDNA that is homologous to the ssDNA from which the nucleoprotein filament is formed. The patterned complex remains stable if ATPγS is used during filament formation.
