# Supplementary material for: Enhancement of RecA-mediated self-assembly in DNA nanostructures through basepair mismatches and single-strand nicks
Source: Sci Rep. 2017 Jan 23;7:41081. doi: 10.1038/srep41081 (PMC5253629; doi:10.1038/srep41081)
Supplement: Supplementary Information [file srep41081-s1.pdf]

Supplementary information

Enhancement of RecA-mediated self-assembly in DNA nanostructures through basepair mismatches and single-stand nicks

Sybilla Louise Corbett, Rajan Sharma, Alexander Giles Davies, and Christoph Wälti*

Sequences

Base pairing sequences are shown in regular text. The mismatched sequences are shown in italics. The triple stranded formation region is emboldened.

100 base sequence
5′ TTT TGA TAG CTT CAA GCC AGA GTT GTC TTT TTC TAT CTA CTC TCA TAC AAC CAA TAA ATG **CTG AAA TGA ATT CTA AGC GGA GAT CGT CTA** GTG ATT TTA A 3′

2 base proximal mismatch
5′ TTT TGA TAG CTT CAA GCC AGA GTT GTC TTT TTC TAT CTA CTC TCA TAC AAC CAA TAA A *CA* **CTG AAA TGA ATT CTA AGC GGA GAT CGT CTA** GTG ATT TTA A 3′

6 base proximal mismatch
5′ TTT TGA TAG CTT CAA GCC AGA GTT GTC TTT TTC TAT CTA CTC TCA TAC AAC CAA *CGA CGT* **CTG AAA TGA ATT CTA AGC GGA GAT CGT CTA** GTG ATT TTA A 3′

8 base proximal mismatch
5′ TTT TGA TAG CTT CAA GCC AGA GTT GTC TTT TTC TAT CTA CTC TCA TAC AAC C *CT GCT CAA* **CTG AAA TGA ATT CTA AGC GGA GAT CGT CTA** GTG ATT TTA A 3′

10 base proximal mismatch
5′ TTT TGA TAG CTT CAA GCC AGA GTT GTC TTT TTC TAT CTA CTC TCA TAC AA *G GTT CGC GGT* **CTG AAA TGA ATT CTA AGC GGA GAT CGT CTA** GTG ATT TTA A 3′

12 base proximal mismatch
5′ TTT TGA TAG CTT CAA GCC AGA GTT GTC TTT TTC TAT CTA CTC TCA TAC *CCG TGT AGT GCC* **CTG AAA TGA ATT CTA AGC GGA GAT CGT CTA** GTG ATT TTA A 3′

15 base proximal mismatch

5′ TTT TGA TAG CTT CAA GCC AGA GTT GTC TTT TTC TAT CTA CTC TCA *ATG TGT AGT GCC GGT* **CTG AAA TGA ATT CTA AGC GGA GAT CGT CTA** GTG ATT TTA A 3′

20 base proximal mismatch
5′ TTT TGA TAG CTT CAA GCC AGA GTT GTC TTT TTC TAT CTA C *CT TCC CCT GTA GTG CCG TTC* **CTG AAA TGA ATT CTA AGC GGA GAT CGT CTA** GTG ATT TTA A 3′

2 base central mismatch
5′ TTT TGA TAG CTT CAA GCC AGA GTT GTC TTT TTC TAT CTA CTC TCA TAC A *TA* CAA TAA ATG **CTG AAA TGA ATT CTA AGC GGA GAT CGT CTA** GTG ATT TTA A 3′

6 base central mismatch
5′ TTT TGA TAG CTT CAA GCC AGA GTT GTC TTT TTC TAT CTA CTC TCA TA *T GTA GT* A TAA ATG **CTG AAA TGA ATT CTA AGC GGA GAT CGT CTA** GTG ATT TTA A 3′

8 base central mismatch
5′ TTT TGA TAG CTT CAA GCC AGA GTT GTC TTT TTC TAT CTA CTC TCA T *CT GTA GTG* TAA ATG **CTG AAA TGA ATT CTA AGC GGA GAT CGT CTA** GTG ATT TTA A 3′

10 base central mismatch
5′ TTT TGA TAG CTT CAA GCC AGA GTT GTC TTT TTC TAT CTA CTC TCA *ACT GTA GTG C* AA ATG **CTG AAA TGA ATT CTA AGC GGA GAT CGT CTA** GTG ATT TTA A 3′

12 base central mismatch
5′ TTT TGA TAG CTT CAA GCC AGA GTT GTC TTT TTC TAT CTA CTC TC *C CCT GTA GTG CC* A ATG **CTG AAA TGA ATT CTA AGC GGA GAT CGT CTA** GTG ATT TTA A 3′

15 base central mismatch
5′ TTT TGA TAG CTT CAA GCC AGA GTT GTC TTT TTC TAT CTA CTC TC *C CCT GTA GTG CCG TT* G **CTG AAA TGA ATT CTA AGC GGA GAT CGT CTA** GTG ATT TTA A 3′

12 base central mismatch – 8 base distance
5′ TTT TGA TAG CTT CAA GCC AGA GTT GTC TTT TTC TAT CTA CT *G CGT ATA TTG GC* A TAA ATG **CTG AAA TGA ATT CTA AGC GGA GAT CGT CTA** GTG ATT TTA A 3′30 base patterning oligomer
5′ CTG AAA TGA ATT CTA AGC GGA GAT CGT CTA 3′

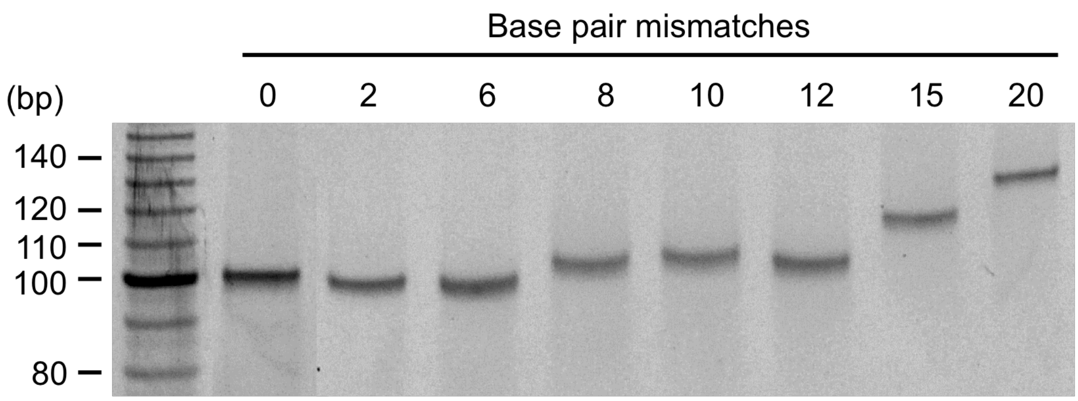

Figure S1: 15% PAGE gel (19:1, 80V, 360 min) showing dsDNA obtained by annealing 100-basepair-long oligonucleotides containing mismatches of 0, 2, 6, 8, 10, 12, 15 and 20 bases located directly adjacent to the nucleoprotein filament assembly site. The bands in the first lane represent a 10 bp DNA ladder.

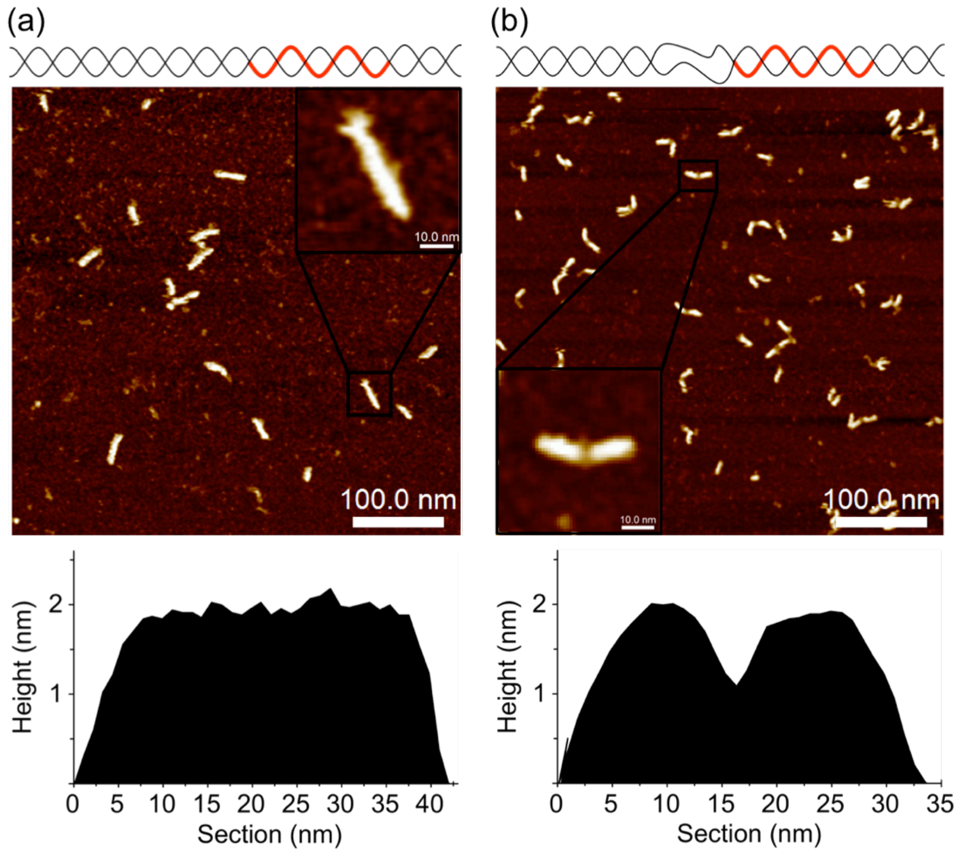

Figure S2: Peak force tapping AFM image of 100 bp dsDNA molecules containing a) fully base paired matching sequence b) a 20 basepair mismatch centered 10 bases away from the

patterning site. Diagrammatic representations of the structures are shown at the top. For the sample height analysis, a single DNA molecule shown in the zoomed inset (10 nm scale bar) was used. The graphs showing the height profiles across a single dsDNA are shown at the bottom.

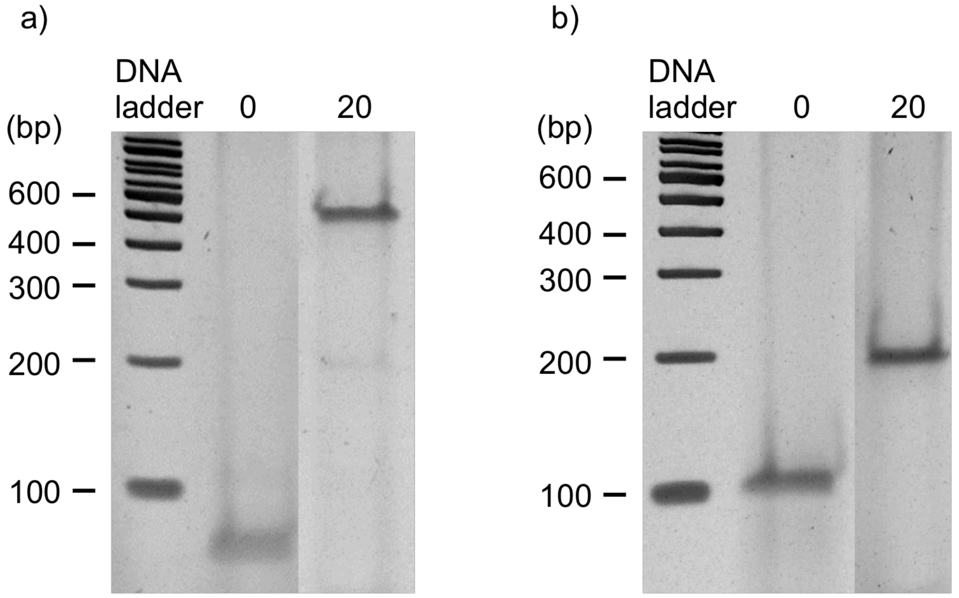

Figure S3: Control results: 15% PAGE gel (19:1, 80V, 360 min) showing a) complete digestion of 100 bp DNA containing fully matched (lane 2) or 20 basepair mismatched sequence (lane 3) following the site-specific patterning of the scaffold with nucleoprotein filaments formed with heat denatured RecA proteins; b) 100 bp DNA containing a fully matched (lane 2) or 20 basepair mismatched sequence (lane 3) following the denaturation of the site-specific patterned complex with Proteinase K. The intermediate XapI digestion step was not performed. The bands in the first lanes represent a 100 bp DNA ladder.

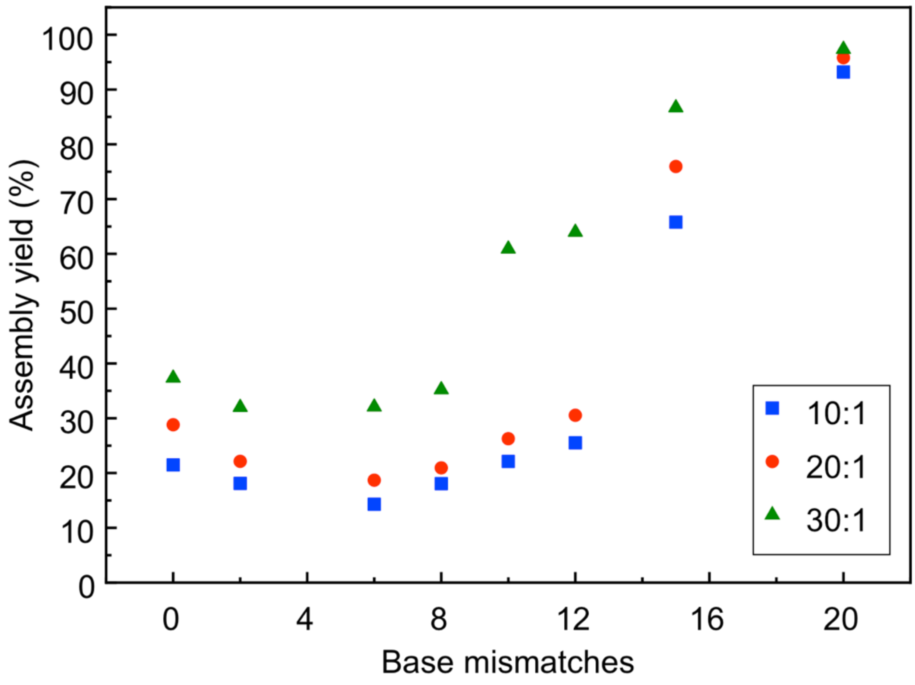

Figure S4: Densitometry analysis showing the effect of the ratio of nucleoprotein filaments to dsDNA scaffold on the yield of nucleoprotein filament-mediated protection from the XapI enzyme digestion for DNA molecules containing 0–20 basepair mismatches, each centered 10 bp away from the patterning site. The results are shown for the assembly carried out at three different ratios of nucleoprotein filament to dsDNA: 10:1, 20:1 and 30:1.

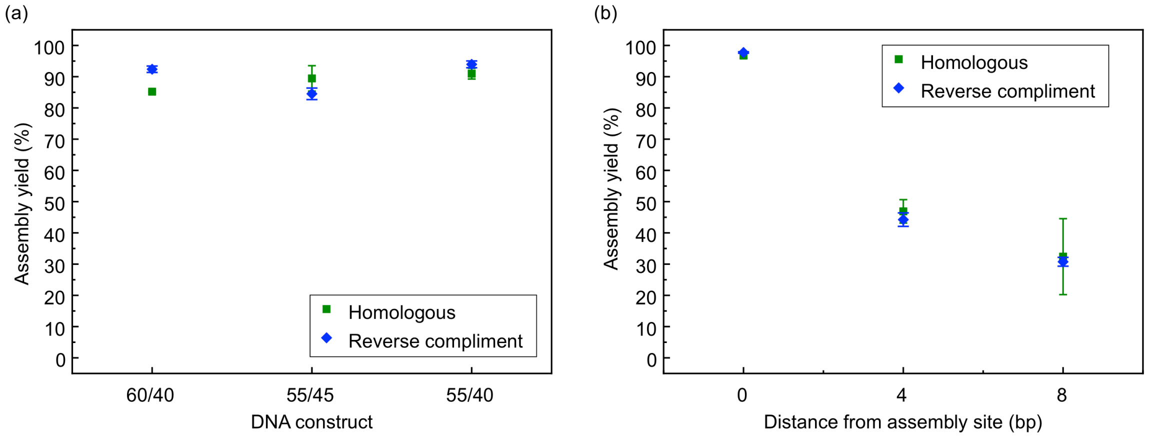

Figure S5: Densitometry analysis showing the comparison of the level of protection for the DNA scaffold achieved from XapI digestion when the scaffold was patterned with 30-

basepair-long nucleoprotein filament with nucleotide sequence matching either the top strand (homologous) or the bottom strand (reverse compliment) of the scaffold. The site for nucleoprotein patterning was maintained 60 bp from the 5' terminal for all the DNA scaffolds. In a) two different 100 bp DNA containing a nick on the top strand located at 60 bp, and 55 bp, and labeled 60/40 and 55/45, respectively from the 5' terminal of the molecule was used. An additional third 100 bp DNA construct where a 5-nucleotide-base gap located 55 bp from the 5' terminal was tested (labeled 55/40); whereas in b) three different 100 bp DNA all containing 12 base mismatched region located at 0, 4 and 8 bases away from the nucleoprotein filament assembly site was used. Error bars show standard error of the mean, n=3.
